# The influence of intergenerational trauma on epigenetics and obesity in Indigenous populations - a scoping review

**DOI:** 10.1080/15592294.2023.2260218

**Published:** 2023-09-26

**Authors:** Krista Schafte, Sean Bruna

**Affiliations:** Department of Anthropology, Western Washington University, Bellingham, WA, USA

**Keywords:** Intergenerational trauma, epigenetics, obesity, Indigenous, motherhood, healing

## Abstract

**Background:** Research has recently begun to examine the potential intergenerational impacts of trauma on obesity.

**Objective:** This scoping review examines the literature on the interactions between intergenerational trauma, epigenetics, and obesity in Indigenous populations. The review was conducted to identify what is known from the literature about how intergenerational trauma may epigenetically influence obesity in Indigenous populations.

**Methods:** Following the PRISMA-ScR guidelines for scoping reviews, online databases were used to identify studies that included discussion of the four focus topics: trauma, epigenetics, obesity, and Indigeneity. The review resulted in six studies that examined those themes. The focus and findings of the selected studies varied from cultural to biological mechanisms and from discussion regarding trauma, epigenetics, obesity, or Indigeneity, but they support three broad statements. First, they support that obesity has genetic and epigenetic factors. Second, intergenerational trauma is prevalent in Indigenous communities. Finally, intergenerational trauma has cultural and biological influences on obesity.

**Conclusions:** Current literature illustrates that intergenerational trauma has behavioural and epigenetic influences that can lead to increased obesity. This scoping review provides a preliminary map of the current literature and understandings of these topics. This review calls for continued studies regarding the connection between trauma, obesity, and epigenetics in Indigenous communities. Future research is vital for practice and policy surrounding individual and communal healing.

## Introduction

Obesity has long been a research topic in Indigenous communities [1]. However, research has only recently begun to examine the potential intergenerational impacts of trauma on obesity [2]. This review examines the literature on intergenerational trauma, epigenetics, obesity, and Indigeneity. These topics, and the interactions between them, are minimally researched. Greater combined understanding in these areas could aid in providing frameworks for awareness and healing. Intergenerational trauma refers to emotional and psychological wounding transmitted across generations [3]. This may include historical trauma, which can be an event or set of events perpetrated on a group of people that share a specific group identity [4], or the cumulative emotional and psychological wounding over a lifespan and across generations [5]. Examining historical trauma can provide a framework for understanding health disparities with psychological, social, and biological mechanisms [5]. Intergenerational trauma can also stem from other traumas such as starvation, malnutrition [6], or maternal stress during pregnancy [7]. Studying these traumas and their mechanisms of transmission can help determine potential causes for health issues and disparities. These studies may also assist in developing frameworks to use in moving towards healing.

The importance of epigenetics within this topic must be illuminated. Definitions of epigenetics are based on current understandings of inheritable patterns of gene expression [8]. Any process that alters gene activity, causes modifications without transforming the DNA sequence, and can be transmitted to daughter cells, are epigenetic processes [9]. Several mechanisms that lead to modifications of gene expression have been revealed, and include acetylation, methylation, phosphorylation, ubiquitylation, and sumoylation [9]. DNA methylation is the addition or removal of a methyl group to a carbon position on a cytosine ring [9,10]. Chromatin modification is another major component of epigenetics: chromatin is the state where DNA is packaged within a cell [11]. Chromatin is composed of histones (proteins) and can be modified by substances such as acetyl groups, enzymes, and microRNAs [9]. This great complexity in how histones can be modified can alter chromatin structure and influence gene expression [9]. These mechanisms are interconnected and can result in activation or deactivation of gene expression [9,11]. Evidence has shown that epigenetic processes are linked with behaviour and mental health, as well as immune system effects and diseases such as cancer [9]. In addition, epigenetic effects have been found in the offspring of parents who have undergone trauma [2]. Recent studies have found that it is possible to reverse some modifications as well, which poses interesting routes for research in the health fields [9]. While research like this is currently expanding on epigenetic models and different classes of modifications [11], this review focuses on epigenetic influences surrounding trauma and health issues that include ‘epigenetic diseases,’ which is where above-mentioned alterations in gene expression can result in health conditions later in life [12].

One of these health issues is obesity, which is recently considered an ‘epigenetic disease’ [12]. Within this perspective, environmental impacts on maternal nutrition during early embryo life leave a ‘nutritional imprint,’ which has long-term effects on the advancement of obesity and other conditions in adulthood [12,13]. The environmental impacts may include parental conditions such as maternal size/obesity, famine during perinatal periods, the use of nutritional supplements, and alcohol or drug abuse [14]. The nutritional imprint from these environmental impacts comprises alterations in physiological processes and programming of gene expression patterns. The specific processes that are modified include ‘embryonic, placental, and foetal growth, organogenesis or regulatory set points for system functions that affect adiposity, where inflammatory and immunologically mediated processes may be involved’ [15]. The programming of these gene expression patterns in embryos can persist into adulthood, contributing to metabolic syndrome features such as abdominal obesity, hypertension, and insulin resistance [15]. Epigenetic marks are present not just for inflammatory processes and adipogenesis but also for energy metabolism [15]. These mechanisms and processes are currently being examined in research: from approximately 760 human genes currently under epigenetic regulation, about 20% of them may be associated with obesity and are defined as epiobesogenes [15]. Considering the epigenetic factors involved in obesity, biological and social scientists should appraise developmental and epigenetic mechanisms as links between these early life environmental factors and adult race-based health disparities in diseases like hypertension, diabetes, stroke, and coronary heart disease [7].

Many health disparities and traumas exist in Indigenous populations. Traumatic experiences for Indigenous people include ‘genocide, ethnocide, forced removal from their homelands, forced removal of children from their homes, and other forced assimilation policies’ that interfere with traditional lifeways [5]. Many Indigenous communities include the loss of language, land, and traditional lifeways as traumas [5]. For American Indians and Alaska Natives, historical traumatic events are combined with high rates of trauma throughout an individual’s life, as well as high rates of chronic stressors, including microaggressions and daily discriminatory events [4]. This trauma, coupled with a lack of access to healthy environments, is often linked to devastatingly high rates of health disparities: poor health in these communities manifests in disproportionately high rates of chronic and communicable diseases [4]. Particularly with cardiometabolic diseases, Indigenous peoples experience the greatest disparity in the United States [5]. There is evidence that adverse exposures epigenetically modify genes associated with cardiometabolic disease risk due to early-life exposures that Indigenous women and children face [16].

Given these disparities within Indigenous communities, this scoping review was completed to examine recent studies investigating the relationships between intergenerational trauma, epigenetics, obesity, and Indigeneity. This review identifies current trends in these studies, the gaps in existing research, and provides recommendations for future research and healing policies.

## Methods

A scoping review was conducted to systematically review existing literature on the relationships between intergenerational trauma, epigenetics, obesity, and Indigeneity. To enhance the formulation and quality of this review, ‘The Preferred Reporting Items for Systematic Reviews and Meta-Analyses Extension for Scoping Reviews’ (PRISMA-ScR) [17] was used (see A1). The protocol for this study was not registered. Since scoping reviews do not focus on quality assessment [18], the studies were not critically appraised.

### Inclusion criteria

The articles selected examined trauma, epigenetics, obesity, and Indigeneity. To be included in the review, articles needed to focus on specific topics (Trauma, Epigenetics, Obesity, and Indigeneity) and their extensions. Peer-reviewed scholarly articles were included if they were: (1) published in English-language journals, (2) empirical human studies, and (3) included discussion on at least two out of the four focus topics (Trauma, Epigenetics, Obesity, and Indigeneity). Selecting articles that mentioned at least two of the four topics ensured that the articles were related and aligned with the purpose of this review, and would discuss interactions between these topics and dimensions. Observational studies of different study designs were included to admit different aspects of measuring and observing the focus topics. These study designs were comprised of cross-sectional studies, cohort studies, and/or qualitative surveys or interviews. Articles were excluded if they did not fit into the conceptual framework of the study. For example, articles that discussed only one of the four topics were not considered to be specific enough to the goal of the review and were therefore excluded. Articles were not included if they were reviews, grey literature, or opinion pieces. Studies were considered if the sample contained both Indigenous and non-Indigenous people, but not all the included studies gathered data on Indigenous people. There was not a time limiter on this scoping review in order to examine any literature on these topics, but the selected studies were published between the period of 2015–2021.

### Data sources and searches

To identify potentially relevant literature, an initial literature search was conducted from 18 January 2022, to 31 January 2022, using combinations of search terms and three electronic bibliographic databases. Search strategies can be found in the Appendix (A2). The search terms were ‘intergenerational trauma’ (alternatives: transgenerational trauma, cross-generational trauma), ‘epigenetics,’ ‘obesity,’ and ‘Indigenous’ (alternatives: American Indian, First Nations, Aboriginal). The electronic bibliographic databases were Western Washington University’s Library OneSearch and Interlibrary Loan and Article Delivery, PubMed, and Google Scholar. These databases were selected due to their availability to the authors and prevalence in the field. The search terms were entered into the databases’ search engines, and subsequent searches were conducted using the alternative search terms. A secondary search using Google Scholar was completed with all the search terms and their alternatives during the screening process. This secondary search was to adjust for potentially missed articles, as the Western Washington University’s Library OneSearch database was limited, and to adjust for articles that may have been published since the first screening step began. Google Scholar offers a ‘digital snowball’ method for scholars to retrieve literature through an organized and instant citation finder [19]. This function was utilized during the secondary search to locate additional literature to use as background or supporting information. The use of a singular search engine as a secondary search saved time during the screening process while scoping for any recently published or missed studies. These search strategies were refined through an iterative process. Because Google Scholar offers this ‘digital snowball’ method and ranks articles that have the most relevancy to the search terms at the top of the search results [19], only the first five pages of Google Scholar were reviewed when it was used.

### Study selection

**Figure 1.**
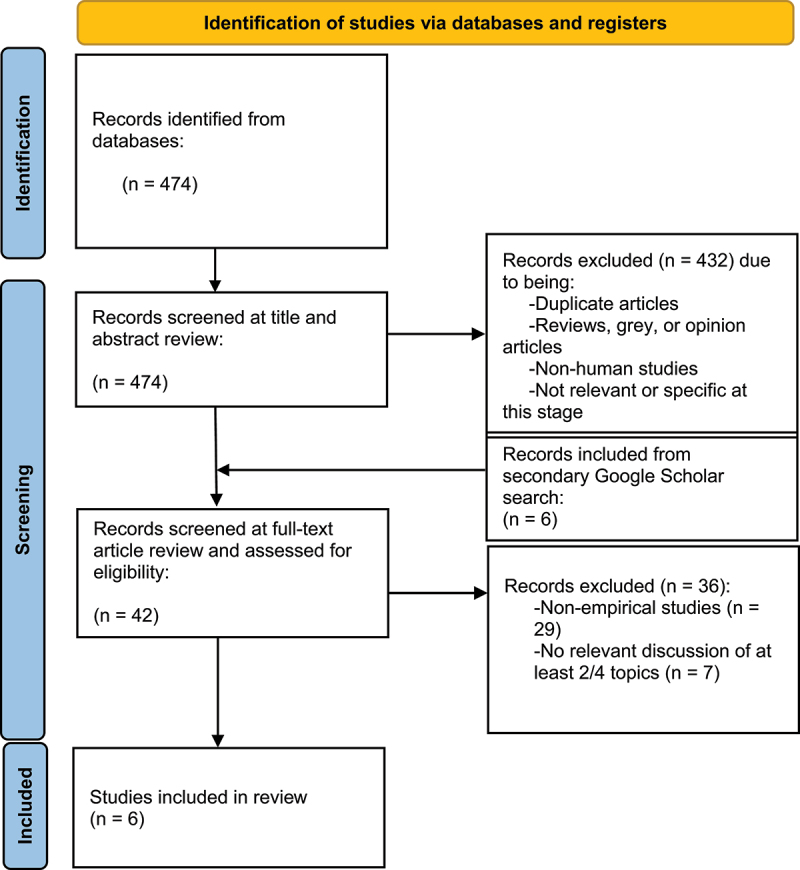
Selection process in PRISMA flow diagram.

Articles were screened using a two-step process: a title and abstract review, and an article review. At each step, the articles were screened for inclusion and exclusion criteria. The initial search resulted in 474 articles. At the title and article review step, articles were excluded if they were duplicates, if they were animal studies, if it was clear that they were reviews, grey literature, or opinion pieces, or if they were not relevant. Relevance at this stage was determined if the title or abstract mentioned at least two out of four of the primary interests. Articles were also excluded if they were not specific to the topics. For example, if an article just mentioned obesity but focused on Type 2 Diabetes instead, it was excluded. This title and abstract review resulted in 36 articles (*n* = 36). In the article review stage, full texts of the articles were examined. Articles were excluded if they were non-empirical studies or if they were not relevant. Relevancy at this stage was determined if the articles fit into the methodological or conceptual framework of the study. For example, if an abstract had mentioned two out of four of the topics but the full text of the article only mentioned it once and conducted no true discussion of the intersection of those topics, it was excluded. At this time, the secondary search was completed using Google Scholar, using the same inclusion and exclusion criteria from the first title and abstract review step. This secondary search identified six additional articles (*n* = 6). These articles were also screened using the same inclusion and exclusion criteria from the first article review step. After reviewing all articles in the article review step (*n* = 42), the article review resulted in six eligible articles to be included in this review (*n* = 6). Of these articles, only one included all four primary interests. The screening process and reasons for exclusion are illustrated in Figure 1 as per the PRISMA-ScR guidelines.

### Data synthesis

Data from each of the included articles were extracted into a spreadsheet and then established in tables for the reporting of data. The choice of tables to display the data was made to facilitate comparison between the reviewed studies. From each study, data was extracted on study characteristics such as study sites, sample populations, and study design (Table 1).

**Table 1.** Characteristics of reviewed studies.

Authors (Year)	Study Site	Years(s) of data collection	Sample Size and Population	Age Range of Sample	Study Design
1- [20]	Ukraine	July-November 2010	45 participants (15 families: survivors of the Holodomor Genocide and their adult children and grandchildren)	86.4, 57.6, 3.3 years (means of each generation)	Qualitative
2- [2]	University of Lethridge, Alberta, Canada	n/a	90 participants (self-identified as First Nations, Inuit, or Métis)	Over 18 (mean: 28 years)	Cross-sectional & Qualitative
3- [21]	Australia	n/a	26 participants (ME [Middle Eastern Mothers])	35–59 years	Qualitative
4- [24]	Mexica	n/a	19 participants (9 Mexican geneticists and 10 medical staff)	n/a	Qualitative
5- [22]	UK	n/a	209 grandmother-mother pairs and 355 grandchildren from Isle of Wight birth cohort	1–26 years	Cohort
6- [23]	Six Nations Reserve and Pigeon Lakes Reserve, Canada	2015–2017	Phase 1 = 37 participants (parents, elders, academic researchers, health service representatives); Phase 2 = 23 participants (community members providing health care services, parents, and community members with Indigenous knowledge)	n/a	Qualitative & 2-phase

Data was extracted on the overall themes of each study (Table 2). This was done in order to analyse and compare the studies’ inclusion of this review’s primary interests and how they approached the themes.

**Table 2.** Themes of reviewed studies.

Authors (Year)	Theme	Obesity	Epigenetics	Trauma	Indigeneity	Intergenerational vs Transgenerational
1- [20]	Behaviorally, Culturally	No	Yes	Yes	No	Both
2- [2]	Behaviorally, Biologically	Yes	Yes	Yes	Yes	Both
3- [21]	Behaviorally, Culturally	Yes	No	Yes	No	Intergenerational
4- [24]	Biologically, Culturally	Yes	Yes	No	Yes	Transgenerational
5- [22]	Biologically	Yes	Yes	No	No	Both
6- [23]	Behaviorally, Culturally	Yes	No	Yes	Yes	Intergenerational

Data was also extracted on the linkages between these themes and major take-aways from each study (Table 3).

**Table 3.** Results of reviewed studies.

Authors (Year)	Impact of Intergenerational Trauma	Linkage between Epigenetics and Obesity	Discussion of Indigeneity	Maternal or Paternal	Future Research
1- [20]	Trauma impacted emotions and inner states and influenced trauma-based coping strategies	Social experiences may affect genetic expression: epigenetics may provide context on the generational transmission of trauma and collective trauma. The impacts found sometimes led to overweight individuals	n/a	Article is not explicit on maternal influence, but only mentions the habits and influences of mothers	Multi-level frameworks of healing
2- [2]	Traumatic stress may be passed down via epigenetic and behavioural pathways. Parental experience in a residential school influenced allostatic load on their offspring	Parental experience in residential schools increased allostatic load on offspring, regardless of if they were raised by these parents	Health and social disparities in Indigenous Canadians are rooted in colonialism, racism, and oppression. Residential schools imparted emotional, physical, and sexual abuse as well as neglect. Repeated or chronic stress is demonstrated by allostasis built on the psychosocial model	Maternal line	Additional research and better understandings of neurobiological and genetic components of trauma stemming from residential schools, and thus health inequalities
3- [21]	Diaspora sometimes resulted in diet acculturation and intergenerational trauma, creating a culture of feeding	n/a	n/a	Mothers and maternal line	Culturally relevant programs, parent workshops, and government initiatives
4- [24]	n/a	Obesity can have epigenetic influences: diet and lifestyle of mothers can shape health and disease outcomes for offspring and future generations; obesity is one example of this	Studies show high levels of genetic markers that are Indigenous American in origin in the mitochondrial DNA of Latin American mestizos, while their Y-chromosome DNA contains high levels of genetic markers that are European in origin. As Indigenous genetic ancestry is linked to obesity, discourse arise centred on racialized and gendered concepts of heredity	The maternal body. Epigenetics can reinforce racialized differences and health inequities, while serving as technologies to reclaim history, culture, and knowledge	Evaluate how Indigenous and maternal bodies are being placed at the centre of health risks linked to obesity
5- [22]	n/a	There is a transgenerational role of grand-maternal BMI on increased grandchild’s BW	n/a	Maternal line	Future studies on the paternal line
6- [23]	Intergenerational trauma has a large influence on community and individual health, and obesity	n/a	Indigenous Canadians have a disproportionate burden of obesity and obesity-related diseases, which could be related to intergenerational trauma exposures. Indigenous ways of life such as traditional knowledge and teachings are synonymous with participation in physical activity and healthy food choices. Cultural continuity is protective in these concerns	Mothers participated in study	Cultural connection and cultural continuity; interventions must be Indigenous-led and multilevel

## Results

### Characteristics of included studies

The reviewed studies were conducted in several countries, encompassing various populations, ethnicities, and generations. The studies examined different kinds of trauma, including individual and collective trauma ranging from genocide, diaspora, residential schools, and loss of Indigenous traditions. The reviewed studies included participants from five countries: Ukraine (*n* = 1), Canada (*n* = 2), Australia (*n* = 1), Mexico (*n* = 1), and the UK (*n* = 1). These articles were published in peer-reviewed journals between 2015 and 2021.

Of the six studies, four used qualitative methods, one used cross-sectional methods in addition to qualitative, and one used a cohort design. Recruitment of the participants included snowball sampling, flyers, and/or social media. Study samples varied greatly, as did the focus of each research study. Not all selected studies incorporated all four primary interests (trauma, epigenetics, obesity, and Indigeneity). No study provided empirical data on the epigenetic relationship or mechanistic pathways between intergenerational trauma and obesity, and instead proposed hypothetical relationships based on other conceptual scholarship.

### Quantitative

The study that used quantitative methods, ‘Maternal Birth Weight and BMI Mediate the Transgenerational Effect of Grandmaternal BMI on Grandchild’s Birth Weight’ by Shen et al, utilized a cohort design [22]. The objective of this study was to examine potential associations between grandmaternal BMI and grandchild’s birth weight, and the possibly mediatory influence of maternal birth weight and BMI [22]. Physical measurements were taken from the Isle of Wight birth cohort in the UK, which included 775 participants (209 grandmother-mother pairs, and 355 grandchildren) [22]. This study included biological focuses on obesity and epigenetics.

### Mixed methods

The study that used mixed methods, ‘The Biological Impacts of Residential Schooling on the Development of Intergenerational Trauma among Indigenous People,’ utilized a cross-sectional design of physical measurements and qualitative surveys [2]. The objective of this study was to examine associations between residential school experiences, adverse childhood experiences, and allostatic load scores among Indigenous students [2]. This data was drawn from 90 participants in Canada that self-identified as First Nations, Inuit, or Métis. This study focused on behavioural and biological influences on obesity, epigenetics, trauma, and Indigeneity.

### Qualitative

The four studies that only used qualitative methods utilized semi-structured interviews. Bezo and Maggi in ‘Living in “survival mode:” Intergenerational transmission of trauma from the Holodomor genocide of 1932–1933 in Ukraine’ investigated the intergenerational impact of the Holodomor Genocide, a starvation event in the Ukraine, by examining survivors of the event. They had 45 participants, consisting of 15 families which included survivors of the genocide and their adult children and grandchildren [20]. This study examined trauma and epigenetics with a behavioural and cultural focus. Hayba et al in ‘Enabling Better Nutrition for Adolescents from Middle Eastern Backgrounds: Semi-Structured Interviews with Parents’ included 26 participants that were Middle Eastern mothers in Australia [21]. The aim of this study was to capture the opinions of the participants regarding the obesity crisis and their influence on adolescent eating behaviours [21]. This study focused on obesity and epigenetics through a behavioural and cultural lens. Saldaña-Tejeda, in ‘Mitochondrial mothers of a fat nation: Race, gender and epigenetics in obesity research on Mexican mestizos,’ interviewed 19 participants which included 9 Mexican geneticists and 10 medical staff [24]. This paper explored scientific and medical discourses on genetics and obesity within the greater context of increasing rates of obesity and diabetes among Mexican mestizos [24]. This study examined obesity, epigenetics, and Indigeneity through a biological and cultural lens. The final study that used qualitative methods was ‘Strategies for Promoting Healthy Nutrition and Physical Activity Among Young Children: Priorities of Two Indigenous Communities in Canada’ by Wahi et al [23]. This study had 60 participants across two phases by employing community engagement workshops before choosing participants. The aim of this study was to support Indigenous communities in identifying strategies for promoting healthy nutrition and activity for adolescents [23]. This study looked at obesity, trauma, and Indigeneity through a behavioural and cultural lens.

#### Relationships between intergenerational trauma, obesity, epigenetics, and Indigeneity

Selected studies explained that traumatic stress was frequently passed down via behavioural and epigenetic pathways. For example, the 1932–1933 Holodomor genocide of Ukrainians not only influenced survivors, but also their adult children and grandchildren [20]. This trauma impacted individual emotions and inner states, as well as affected trauma-based coping strategies. Some of the trauma-based coping strategies included risky health behaviours, stockpiling of food, overemphasis on food and overeating, and extreme reverence for food [20]. This collective trauma, as well as the intergenerational transmission of it, ultimately impacts the family and community even beyond the individual [20]. More than half of the participants in Chief Moon-Riley’s study felt that the parenting they received when they were young was negatively impacted by the experience of their family members who attended residential schools [2]. Parental experience in residential schools influenced allostatic load on their offspring [2]. Both experiences are theorized to be traumatic stress passed down via both behavioural and epigenetic pathways [2].

One study found that diaspora may have resulted in not only diet acculturation but also intergenerational trauma, creating a culture of ‘feeding’ that influenced the experience of offspring from Middle Eastern backgrounds [21]. Some first-generation immigrants associated overfeeding as an ‘act of love and care’ [21], impacting the eating and feeding behaviours of their children and grandchildren. A few parents reflected on this and posited that the culture might stem from a psychological need for their parents to compensate for intergenerational trauma, as many were fleeing poverty, war, and violence [21]. Intergenerational trauma was also found to have a significant influence on the community and individual health in Six Nations and Pigeon Lakes Reserves in Canada [23].

Four studies stated that transmissions of intergenerational trauma influenced obesity through epigenetic changes. Obesity can have epigenetic influences, and the diet and lifestyle of mothers can shape health and disease outcomes for offspring and future generations [24]. Additionally, a transgenerational role of grand-maternal BMI was found to increase the grandchild’s body weight [22]. The social experiences of survivors and offspring of the Holodomor genocide may affect genetic expression, and epigenetics may provide context on the transmission of individual and collective trauma [20]. The impacts of this experience were found to sometimes result in overweight individuals [20]. Parental experience in residential schools was found to increase the allostatic load on offspring, regardless of if they were raised by these parents [2], suggesting that epigenetic pathways were involved. Allostasis is the method through which the body deals with stress, and this stress can influence physiological adaptations; allostatic load is the result of ‘being forced to adapt to adverse psychosocial or physical situations’ [2]. The biomarkers for determining allostatic load in Chief Moon-Riley’s study include body mass index, waist circumference, blood pressure, cortisol awakening response, and dehydroepiandrosterone sulphate, several of which biomarkers are also indicators of obesity [2]. This study builds on previous research by highlighting that it is most likely the maternal childhood experience within residential schools that impacts the allostatic load of offspring instead of the impacts of residential schooling on maternal behaviour to future offspring [2]. This further illustrates potential epigenetic pathways that influence obesity from trauma. While the work of allostasis and allostatic load complement the work within the field of epigenetics, the epigenetic process could not be directly examined in Chief Moon-Riley’s study: blood samples and DNA information were not collected [2].

One study stated that for the mitochondrial DNA of Latin American mestizos, there are high levels of genetic markers that are Indigenous American in origin, while their Y-chromosome DNA contains high levels of genetic markers that are European in origin [24]. As Indigenous genetic ancestry is linked to obesity, discourse arises centred on racialized and gendered concepts of heredity [24].

Another pattern within these selected studies is the focus on the parental method of transmission of trauma, whether epigenetically or behaviourally. When investigating the transmission of trauma or its influence on obesity, the studies highlighted maternal influence rather than paternal. Chief Moon-Riley’s research found that participants who had a mother attend residential school were five times more likely to have an allostatic load score in the mid-range than the low-range compared to participants who did not have a mother attend residential school: this association was not found for fathers who attended residential school [2]. The study by Shen et al. focused only on the maternal line, finding that a larger grand-maternal BMI indirectly increased the grandchild’s body weight via maternal body weight and BMI [22]. In Australia, only mothers were interviewed [21], and at Six Nations Reserve and Pigeon Lakes Reserve in Canada, only mothers were mentioned to participate in the study, not fathers [23]. Bezo and Maggi’s article on the Holodomor genocide was not explicit on maternal influence but only mentioned the habits and influences of mothers [20]. Saldaña-Tejeda’s article primarily focused on maternal bodies and inheritance while critiquing assumptions and implications often made in genetic and epigenetic studies; that the uteri of young mothers determine the fate of their bloodlines for generations, creating gendered notions of heredity [24].

## Discussion

This scoping review reveals several key features of the current literature on intergenerational trauma, obesity, epigenetics, and Indigeneity. First, the literature supports that obesity has genetic and epigenetic factors. Second, intergenerational trauma is prevalent in Indigenous communities. Finally, intergenerational trauma has cultural and biological influences on obesity.

Epigenetics is potentially helpful in determining adverse health effects and the causes of those effects. It can also offer a compelling framework to ‘link social pasts with biological presents, providing a culturally relevant way of understanding the embodied transmission of trauma and ill health across generations’ [25]. However, there are limitations of epigenetic studies, and there is difficulty in determining transgenerational epigenetic inheritance in humans. Researchers must rule out genetic, ecological, and cultural inheritance, identify the responsible epigenetic factor in the germ cells, and demonstrate that the factor in the germ cells is responsible for the phenotypic effect in the next generation [26]. Considering the long lifespans of humans and the difficulty of manipulating DNA methylation and histone modification, determining and proving epigenetic inheritance across several generations is challenging.

Although the selected studies focused on the maternal line, there are some implications of inheritance from the paternal line. Studies not in the review have found linkages between cardiovascular mortality and a father’s nutritional status, and an increase in body mass index was found in F2 of famine-exposed fathers, but not famine-exposed mothers [12]. While there is currently less research and evidence for paternal inheritance than maternal inheritance in humans, it is a growing area of study, and several animal studies have been completed. For example, one study found that in a mouse model of paternal postnatal trauma, transgenerational transmission of behavioural and metabolic phenotypes existed up to the 4th generation [27]. Another found that sperm RNA microinjection experiments may suggest that within metabolic pathologies of mice, sperm RNAs are sufficient for establishment but not for long-term epigenetic inheritance [28]. The data from an additional study indicated that induced metabolic phenotypes may propagate for a generation ‘beyond any direct exposure to an inducing factor,’ and that the inheritance likely occurs via sperm noncoding RNA [29].

Many human epigenetic studies have found evidence of maternal transmission rather than paternal. This can affect motherhood, including what motherhood means for individuals and how motherhood is viewed within epigenetics and society. Some scientists in the field believe that epigenetics is progressive and transformative, and some think it is heading towards biomedicalization and victim blaming [25]. Social scientists have raised concerns about the possibility of epigenetics to enhance blame on mothers for the well-being of their children, which would shift ‘responsibility away from the societal factors that are far beyond maternal control’ [25]. An increase in surveillance and governmentality may heighten this burden and blame on mothers as well [30]. In addition, readings of epigenetics may lead to determinism by formulating the perception that an individual’s health and behaviour are destined by epigenetic factors [31].

Intergenerational trauma and its influence on obesity, through both cultural and biological mechanisms, is especially relevant within Indigenous populations. Indigenous Canadians have several health and social disparities and disproportionate rates of chronic disease [2], including a disproportionate burden of obesity and obesity-related diseases, which could be related to intergenerational trauma exposures [23]. These disparities are often rooted in oppression, discrimination, systematic racism, and colonialism [2]. One example of oppression and trauma within Indigenous Canadian communities is residential schools. These schools imparted emotional, physical, and sexual abuse and neglect of Indigenous children [2]. Repeated or chronic stress from traumas like colonialism and residential schooling have been found to influence allostasis and allostatic load, built on the psychosocial model [2].

Indigenous ways of life, such as traditional knowledge and teachings, are synonymous with participation in physical activity and healthy food choices [23]. When there has been a loss of connection to Indigenous traditions and teachings, these traditional activities have been impacted [23], further disrupting healthy behaviours and adding to health disparities.

Epigenetics as a field and public concept could intensify existing pressure on Indigenous parents. This burden includes pressures to ‘overcome the structural barriers their children face and ensure positive epigenetic exposures’ [25]. Thus, these pressures and deterministic positionings are often racialized, as well as gendered [25]. To mediate this considering biological research that addresses social issues, it is crucial that biological research invokes expertise from the social sciences and humanities in order to refine research questions and interpret results [31]. While consideration of these notions is vital, epigenetics does have great potential. It has been proposed as a science that has the capacity to assemble political will towards ‘remedying social inequities and their health effects’ [25]. Epigenetics can trace many of these health disparities to the result of colonization and its aftermath, a ‘colonial health deficit’ [25]. Senior Indigenous researchers have also drawn on epigenetic discourses to discuss how past experiences of trauma can be transmitted to Indigenous children as ‘communal wounds’ [25]. While there is great potential for the field of epigenetics, there is also potential for harm, particularly in minority communities.

The selected studies called for future research on epigenetic studies and examined the best modes of healing regarding trauma and obesity. None of the studies provided empirical data on the epigenetic relationship or mechanistic pathways between intergenerational trauma and obesity: many of the theories on these epigenetic pathways in the included studies were derived from background information, and thus more quantitative studies regarding these epigenetic pathways are needed. While several conceptual reviews and theories exist [7, 16, 32, 33], the results of this literature review found only one empirical study investigating intergenerational trauma, epigenetics, and obesity within Indigenous communities. Minimal research is present on the relationships and intersections between these topics. However, some research, comprising of some of the included studies, does focus on the influence of trauma on obesity through behavioural and/or cultural mechanisms. Trauma can influence behaviour and thus how an individual or culture raises their children. This instilled behaviour can lead to relationships surrounding food and health that affect or influence obesity. Future research within these fields is necessary to consider the relationships between these aspects and the historical and future implications of data.

The authors of included studies called for additional research to lead to a better understanding of neurobiological and genetic components of trauma, and thus health inequalities among Indigenous groups [2]. To examine these mechanisms, authors also urged for future studies on the paternal line [22]. Moving beyond research, there needs to be multilevel healing frameworks concerning collective trauma and its intergenerational transmission; these frameworks would include the individual, the family, and the greater community [20]. Research findings would also support the development of locally designed and culturally relevant programmes, parent workshops, and government initiatives. Programmes that are culturally relevant are consistently advocated for by researchers [21]. The programmes should take into consideration experiences that are unique to immigrant populations, including experiences such as diet acculturation, food insecurity, and traditional feeding practices [21]. Cultural connection and cultural continuity should be focused on, and interventions within Indigenous communities must be Indigenous-led and multilevel [23].

### Limitations

This scoping review has some limitations. Only three databases were utilized, and one of them was a university database. A non-affiliate of the university would not be able to access that database. Additionally, the authors were unable to examine some literature that was not open access, so there is the potential for missed studies. The search terms ‘Native American’ and ‘native’ were missed in the search strategy of this review, so if additional scoping reviews or systematic reviews are conducted, the search terms should be added as alternative terms to account for additional articles with those key terms. Also, the use of a pre-written search strategy would have made the search process faster and more easily repeatable. Including filters at the beginning of the search steps instead of towards the end would have aided in this as well. For example, adding a human studies filter in the initial screening. Finally, it is also important to note that identifying gaps in the current literature through this scoping review does not necessarily describe gaps where the research is of poor quality, since scoping reviews do not typically perform quality assessment [18].

### Conclusion

The findings from this scoping review demonstrate that the current literature theorizes that intergenerational trauma can influence obesity. However, there is limited research on the epigenetic mechanisms of these influences, and how these topics impact Indigenous communities and individuals. This scoping review displays current literature and research and highlights the future research that is needed within these fields to consider the relationships between these aspects and the historical and future implications of data. Animal research on possibilities of adaptive mechanisms like reversion of obesity should continue, as well as research on the human implications of these results. It is essential to examine different kinds of trauma, such as colonial trauma, malnutrition, and adverse childhood experiences, and their effects. Connected with these ideas, protective factors for both obesity and trauma are major considerations for future research. It is also necessary for the completion of more research on the influence of the field of epigenetics on the experience of motherhood. Additional research on the experience of people with obesity in society, especially within Indigenous communities, is another significant operation. It is vital that research in Indigenous communities is ethical and done in conjunction with Indigenous researchers and community members. Relatedly, it is paramount that the development of any health programmes intended to mitigate obesity crises in Indigenous communities are made with Indigenous peoples and are multi-level and culturally relevant.

To design future frameworks of healing, it is crucial to understand the interactions between Indigeneity, trauma, epigenetics, and obesity. These frameworks and programmes may include culturally relevant programmes, parent workshops, and government initiatives. More epigenetic research and research on the experiences of individuals will solidify these understandings and aid in the design of these programmes. This scoping review aids in solidifying connections between the four topics and their dimensions. It helps to bring attention to and calls for more understanding in health care regarding working with individuals with obesity or trauma. While this scoping review provides a preliminary map on the current literature and understandings of these topics, more focused systematic reviews will aid in appraising the quality of additional research and the validity of the results. This will make recommendations for practice and policy relevant to a greater extent.

## Data Availability

The authors confirm that the data supporting the findings of this study are available within the article [and/or] its supplementary materials.

## Appendix

A1. Preferred reporting items for systematic reviews and meta-analyses extension for Scoping Reviews (PRISMA-ScR) checklist

File 2. Search strategies

Databases: Google Scholar, PubMed, and Western Washington University’s Library OneSearch

Search dates: 18 January 2022, to 31 January 2022

1. ‘Intergenerational trauma’ AND ‘epigenetics’ AND ‘obesity’ AND ‘Indigenous’
2. ‘Intergenerational trauma’ AND ‘obesity’ AND ‘epigenetics’ AND Indigenous OR American Indian OR First Nations OR Aboriginal
3. Intergenerational trauma OR transgenerational trauma OR cross-generational trauma, ‘epigenetics’ AND ‘obesity’ AND Indigenous OR American Indian OR First Nations OR Aboriginal

A2. Search Strategies

**Table:** Preferred Reporting Items for Systematic reviews and Meta-Analyses extension for Scoping Reviews (PRISMA-ScR) Checklist.

SECTION	ITEM	PRISMA-ScR CHECKLIST ITEM	REPORTED ON PAGE #
**TITLE**			
Title	1	Identify the report as a scoping review.	1
**ABSTRACT**			
Structured summary	2	Provide a structured summary that includes (as applicable): background, objectives, eligibility criteria, sources of evidence, charting methods, results, and conclusions that relate to the review questions and objectives.	1
**INTRODUCTION**			
Rationale	3	Describe the rationale for the review in the context of what is already known. Explain why the review questions/objectives lend themselves to a scoping review approach.	2
Objectives	4	Provide an explicit statement of the questions and objectives being addressed with reference to their key elements (e.g., population or participants, concepts, and context) or other relevant key elements used to conceptualize the review questions and/or objectives.	5
**METHODS**			
Protocol and registration	5	Indicate whether a review protocol exists; state if and where it can be accessed (e.g., a Web address); and if available, provide registration information, including the registration number.	5
Eligibility criteria	6	Specify characteristics of the sources of evidence used as eligibility criteria (e.g., years considered, language, and publication status), and provide a rationale.	6
Information sources*	7	Describe all information sources in the search (e.g., databases with dates of coverage and contact with authors to identify additional sources), as well as the date the most recent search was executed.	6
Search	8	Present the full electronic search strategy for at least 1 database, including any limits used, such that it could be repeated.	6
Selection of sources of evidence†	9	State the process for selecting sources of evidence (i.e., screening and eligibility) included in the scoping review.	7
Data charting process‡	10	Describe the methods of charting data from the included sources of evidence (e.g., calibrated forms or forms that have been tested by the team before their use, and whether data charting was done independently or in duplicate) and any processes for obtaining and confirming data from investigators.	8–9
Data items	11	List and define all variables for which data were sought and any assumptions and simplifications made.	8–9
Critical appraisal of individual sources of evidence§	12	If done, provide a rationale for conducting a critical appraisal of included sources of evidence; describe the methods used and how this information was used in any data synthesis (if appropriate).	N/A
Synthesis of results	13	Describe the methods of handling and summarizing the data that were charted.	8
**RESULTS**			
Selection of sources of evidence	14	Give numbers of sources of evidence screened, assessed for eligibility, and included in the review, with reasons for exclusions at each stage, ideally using a flow diagram.	Supplementary materials, Fig. 1
Characteristics of sources of evidence	15	For each source of evidence, present characteristics for which data were charted and provide the citations.	9–11
Critical appraisal within sources of evidence	16	If done, present data on critical appraisal of included sources of evidence (see item 12).	N/A
Results of individual sources of evidence	17	For each included source of evidence, present the relevant data that were charted that relate to the review questions and objectives.	11–14
Synthesis of results	18	Summarize and/or present the charting results as they relate to the review questions and objectives.	Supplementary materials, Tables 1, 2, & 3
**DISCUSSION**			
Summary of evidence	19	Summarize the main results (including an overview of concepts, themes, and types of evidence available), link to the review questions and objectives, and consider the relevance to key groups.	14
Limitations	20	Discuss the limitations of the scoping review process.	19
Conclusions	21	Provide a general interpretation of the results with respect to the review questions and objectives, as well as potential implications and/or next steps.	19–20
**FUNDING**			
Funding	22	Describe sources of funding for the included sources of evidence, as well as sources of funding for the scoping review. Describe the role of the funders of the scoping review.	20

JBI = Joanna Briggs Institute; PRISMA-ScR = Preferred Reporting Items for Systematic reviews and Meta-Analyses extension for Scoping Reviews.

*Where *sources of evidence* (see second footnote) are compiled from, such as bibliographic databases, social media platforms, and Web sites.

†A more inclusive/heterogeneous term used to account for the different types of evidence or data sources (e.g., quantitative and/or qualitative research, expert opinion, and policy documents) that may be eligible in a scoping review as opposed to only studies. This is not to be confused with *information sources* (see first footnote).

‡The frameworks by Arksey and O’Malley (6) and Levac and colleagues (7) and the JBI guidance (4, 5) refer to the process of data extraction in a scoping review as data charting.

§The process of systematically examining research evidence to assess its validity, results, and relevance before using it to inform a decision. This term is used for items 12 and 19 instead of ‘risk of bias’ (which is more applicable to systematic reviews of interventions) to include and acknowledge the various sources of evidence that may be used in a scoping review (e.g., quantitative and/or qualitative research, expert opinion, and policy document).

From: Tricco AC, Lillie E, Zarin W, O’Brien KK, Colquhoun H, Levac D, et al. PRISMA Extension for Scoping Reviews (PRISMAScR): Checklist and Explanation. Ann Intern Med. 2018;169:467–473. doi: 10.7326/M18–0850.
